# An *SS18::NEDD4* cutaneous spindled and epithelioid sarcoma: An hitherto unclassified cutaneous sarcoma, resembling epithelioid sarcoma with aggressive clinical behavior

**DOI:** 10.1002/gcc.23071

**Published:** 2022-06-15

**Authors:** Ashley Patton, Steve Oghumu, O. Hans Iwenofu

**Affiliations:** ^1^ Department of Pathology and Laboratory Medicine The Ohio State University Wexner Medical Center Columbus Ohio; ^2^ The James Comprehensive Cancer Center The Ohio State University Columbus Ohio

**Keywords:** cutaneous spindled and epithelioid sarcoma, molecular genetics, *SS18::NEDD4* gene fusion

## Abstract

*SS18::SSX* gene fusions as a result of t(X,18)(p11;q11) have only been described in synovial sarcoma (SS). Recently, an *SS18::NEDD4* gene fusion was identified in a single case of primary renal SS exhibiting a hypocellular and myxoid morphology. Herein, we report a case of an unclassified malignant cutaneous spindled and epithelioid neoplasm in a 60‐year‐old female that resembled an epithelioid sarcoma (ES) and harbored a rare *SS18::NEDD4* gene fusion. Briefly, the patient presented with a progressively growing cutaneous mass involving the volar aspect of right hand, warranting an amputation. Histologic sections revealed a cutaneous ulcerative neoplasm composed of spindled and epithelioid cells, bearing a certain semblance to ES, with diffuse invasion into the subcutis and skeletal muscle. Coagulation tumor necrosis and mitotic figures were present. By immunohistochemistry, the tumor cells were positive for keratins (AE1/3 and cam5.2), vimentin, CMYC, BCL2, p53, smooth muscle actin (focal), and TLE1 (multifocal) and negative for p40, p63, CK5/6, CK7, CK20, CD56, CD31, CD34, ERG, desmin, SMMS, H‐Caldesmon, myogenin, and S‐100. Expression of INI1 stain was retained. The unusual histomorphology and inconclusive immunophenotypic profile lead to next‐generation sequencing identifying an *SS18::NEDD4* gene fusion with genomic coordinates 5′‐*SS18* (ex1‐9 NM_005637)‐*NEDD4* (ex14‐29 NM_006154). Fluorescence in situ hybridization confirmed *SS18* gene rearrangement. Within 2 years, the patient developed widespread metastatic disease. Despite aggressive multimodality treatment, the patient succumbed to disease. In summary, we report a unique case of previously unclassified cytokeratin positive malignant cutaneous spindled and epithelioid sarcoma with aggressive behavior, harboring an *SS18::NEDD4* fusion.

## 1 INTRODUCTION

The *SS18::NEDD4* fusion gene has been recently identified in only one case of primary renal synovial sarcoma (SS) by Argani et al.^1^ Although not yet fully characterized, this gene fusion was reported in a variant of SS, an entity most notably characterized by the t(X;18)(p11;q11) translocation resulting in *SS18* gene rearrangements involving fusion partners *SSX1* in two‐thirds of SS, *SSX2* in one‐third, and rarely *SSX4*.^2^, ^3^, ^4^, ^5^

The detection of *SS18::SSX1/2/*4 fusion gene is considered the gold standard in the diagnosis of SS. However, the increasing use of next‐generation sequencing (NGS), in modern clinical molecular laboratories has enabled the discovery of many novel gene fusions well as molecular pleiotropy in both solid organ and hematologic malignancies. Consequently, the detection of *SS18* rearrangements in entities other than SS has been reported. Specifically, *SS18* rearrangements involving the *MEF2C* and *MEF2D* partner genes, resulting in *MEF2C::SS18* and *MEF2D:SS18* gene fusions, have been identified in microsecretory adenocarcinoma and the B‐cell acute lymphoblastic leukemia, respectively. However, both entities, microsecretory adenocarcinoma of the salivary gland, and acute B‐cell lymphoblastic leukemia, arise from different cellular lineages and display unique histomorphology and clinical courses.^6^, ^7^ Furthermore, the translocation resulting in *CRTC::SS18* has been identified in an undifferentiated small round cell sarcoma with aberrant *ALK* expression. Other undifferentiated round cell sarcomas with novel *SS18‐POU5F1* have also been described previously.^8^ The heterogeneity in histomorphology of tumors harboring *SS18* rearrangements highlights the potential cell‐specific variability of perturbations in BAF regulation, a chromatin remodeling complex that functions to maintain balance in both gene activation and repression.

Herein, we describe an aggressively behaving malignant cutaneous spindled and epithelioid sarcoma harboring an *SS18::NEDD4* gene fusion in a 60‐year‐old female. To date, the *SS18::NEDD4* fusion has only been identified in one case of primary renal SS.^1^ To the best of our knowledge, this index case represents the first report *of SS18::NEDD4* gene fusion in an unclassified sarcoma, other than SS.

## 2 MATERIALS AND METHODS

### 2.1 Immunohistochemistry

Immunohistochemical stains using the following commercially available antibodies were performed using the Leica Bond III autostainers (Leica Biosystems): AE1/E3 (Dako, AE1&AE3, 1:1200), CK7 (Dako, OV‐TL‐12/30, 1:600), CK20 (Dako, Ks20.8, 1:200), CAM 5.2 (BD, CAM5.2, 1:80), vimentin (Dako, V9, 1:1800), BCL‐2 (Dako, 124, 1:1200), smooth muscle actin (Dako, 1A4, 1:600), P63 (Biocare, 4A4, 1:300), Desmin (Dako, D33, 1:200), S‐100 (Dako, R poly, 1:2000), INI1 (BD, BAF47, 1:2000), CD56 (Dako, 123C3, 1:100), TLE1 (BioSB, 1F5, 1:50), P40 (Biocare, BC28, 1:150), P53 (Dako, DO‐7, 1:1000), CMYC (Cell Marque, EP121[Y69], 1:100), ERG (BioCare M, 9FY, 1:50), CD34 (Cell Marque, QBEnd/10, 1:400), CD31 (Dako, JC 70A, 1:400), H‐caldesmon (Biogenex, clone‐h‐CD, 1:500), smooth muscle myosin heavy chain, SMMS (Cell Marque, clone‐SMMS‐1, 1:800), and myogenin (Cell Marque/F5D, 1:200). All protocols were developed by and performed at the OSU Wexner Medical Center Histology Laboratory, 680 Akerman Road, Columbus, OH 43210. All positive and negative controls showed appropriate staining.

### 2.2 Next‐generation sequencing

NGS was performed using the FoundationOne Heme assay (Foundation Medicine, Inc. Cambridge, MA). Total nucleic acid, DNA, and RNA, was extracted from visible tumor in 4‐μm sections of formalin‐fixed paraffin‐embedded tissue. Comprehensive genomic profiling was performed using FoundationOne Heme® (F1H), a clinical‐grade, high‐throughput, hybridization capture‐based NGS assay for targeted sequencing of all exons of 406 genes, and RNA sequencing of 265 genes. F1H methods used have been previously reported in detail.^9^ Sequences were analyzed for genomic alterations including short variant alterations, copy number alterations, and gene rearrangements as described.^10^

### 2.3 Fluorescence in situ hybridization

Fluorescence in situ hybridization (FISH) analysis was performed on a 4‐μm unstained section of representative formalin‐fixed, paraffin‐embedded tissue using a commercially available LSI break‐apart probe set (Vysis). The LSI SS18 Dual Color, Break Apart Rearrangement Probe consists of a mixture of two FISH DNA probes. The first probe, an ~650 kb probe labeled in SpectrumOrangeTM, extends distally from the *SS18* gene. The second probe labeled in SpectrumGreenTM lies 3′ or proximal to the *SS18* gene and is approximately 1040 kb in length. Hybridization signals were assessed in 200 interphase nuclei with strong, well‐delineated signals. Only nonoverlapping tumor nuclei with a complete set of signals were scored. The cut‐off level for scoring aberrations was 15% abnormal nuclei for the dual‐color *SS18* FISH probe system.

## 3 RESULTS

### 3.1 Case report

**FIGURE 1:**
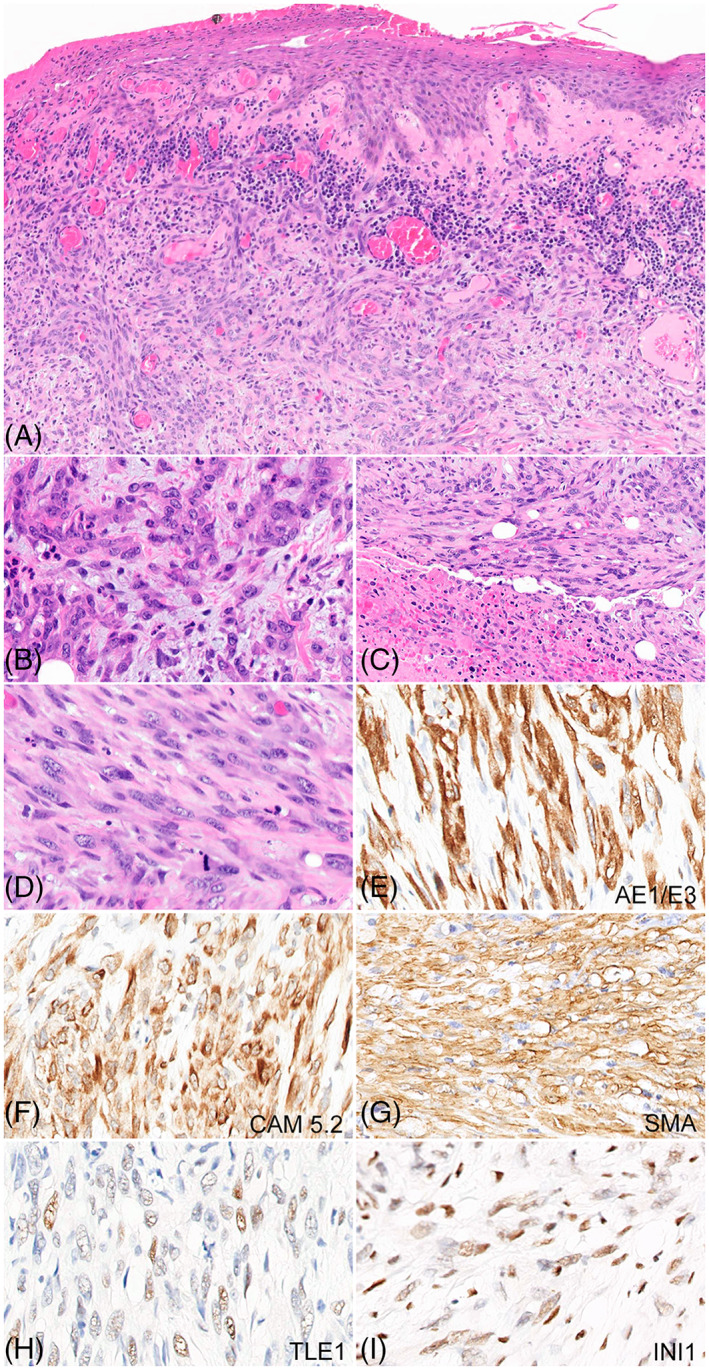
Histopathological features of the index case and immunohistochemical profile. (A) An ulcerative malignant cutaneous lesion with involvement of the dermis and subcutaneous tissue (×40). (B–D) Highlight the dominant histopathological features of epithelioid (B) and spindled phenotype (C–D) in the tumor cells, exhibiting nucleocytoplasmic pleomorphism, mild nuclear hyperchromasia, and mitotic activity [×400) (B&D)]. Coagulative tumor necrosis can be appreciated in panel C (×200). The lesional cells show diffuse and strong positivity for AE1/AE3, CAM 5.2, and SMA, and multifocal positivity (40% overall) for TLE‐1 [(×400) (G–I)]. (J) Retained expression of INI1 in lesional cells (×400).

The patient was a 60‐year‐old female who presented to an outside institution with a mass located on the volar aspect of the right hand. An excisional biopsy was performed to yield an ulcerative cutaneous malignant neoplasm composed of fascicles and nodular aggregates of malignant spindled and epithelioid cells within a hyalinized stroma dissecting through sclerotic collagen bundles, punctuated by confluent zones of coagulative tumor necrosis (Figure 1). The tumor cells manifest nucleocytoplasmic pleomorphism and contained vesicular chromatin with irregular nuclear contours, pinpoint nucleoli and indistinct palely eosinophilic cytoplasm, and brisk mitotic rate (Figure 1). By immunohistochemistry, the tumor cells were positive for keratins (AE1/E3 and Cam5.2), vimentin, p53, smooth muscle actin (SMA), TLE1 (multifocal, 40% overall), CMYC, BCL2 (focal), and negative for p40, p63, and S‐100. The INI1 stain was strongly and diffusely positive, retained in approximately 95% of neoplastic cells.

Given the expression of smooth muscle actin in the tumor cells, we entertained the possibility of a cutaneous myoid/myogenic sarcoma with aberrant cytokeratin expression, but additional myogenic markers including smooth muscle heavy chain myosin (SMMS), H‐caldesmon, desmin, and myogenin were negative, significantly diminishing that diagnostic consideration. Furthermore, we considered the possibility of a cutaneous malignant vascular neoplasm, but again the vascular markers including ERG, CD31, and CD34, were all negative. The histomorphology appeared somewhat reminiscent of an epithelioid sarcoma and was initially labeled as malignant keratin‐positive spindled and epithelioid neoplasm, resembling epithelioid sarcoma. The neoplasm involved the surgical margins requiring multiple attempts for surgical re‐excision. A right forearm amputation was ultimately performed with axillary dissection. Examination of the surgical amputation revealed a 6.7 cm mass, recapitulating the histopathological features previously seen on excisional biopsy. All examined lymph nodes were negative for metastatic disease.

**FIGURE 2:**
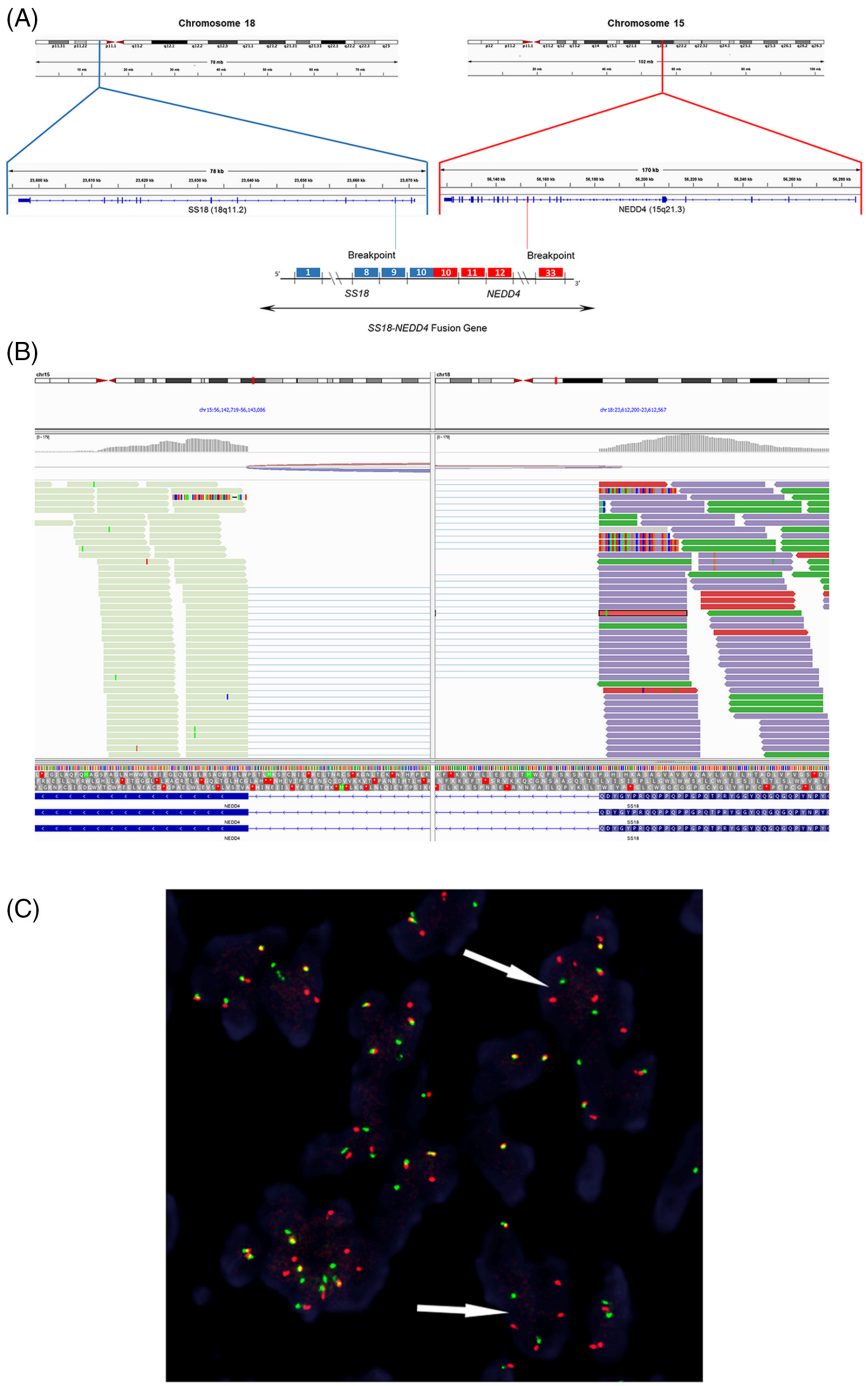
*SS18::NEDD4* fusion identified by next‐generation sequencing (NGS) and confirmed by FISH. Schematic showing the *SS18::NEDD4* gene fusion identified by NGS (A, B). *SS18* break‐apart FISH (×400) demonstrating split orange (5′*SS18*) and green (3′*SS18*) signals indicative of a rearrangement of the *SS18*(18q11.2) locus (C).

One year following amputation of the primary lesion, surveillance positive emission tomography (PET) scan showed bilateral pulmonary nodules biopsied to reveal metastatic disease for which chemotherapy was initiated. One year following neoadjuvant therapy, metastatic lesions were identified within the brain by magnetic resonance imaging (MRI) requiring surgical resection and radiation therapy. Progression of pulmonary metastases was identified on computed tomography (CT) scan as well as newly identified metastatic lesions to the liver and kidney. Soft tissue subcutaneous nodules were also identified along the right and left anterior abdominal wall. Wide local excision of the subcutaneous nodules was performed to reveal three discrete, depressed, ulcerated lesions measuring 2.6 × 0.8 cm, 2.4 × 1.5 cm, and 4.6 × 4.1 cm. The histomorphology and immunophenotype were similar to the previous amputation specimen, confirming widely metastatic disease. The unusual histomorphology and inconclusive immunophenotypic profile, prompted NGS to further characterize the lesion. Results from the NGS panel identified an *SS18::NEDD4* fusion gene with genomic coordinates 5′‐SS18 (ex1‐9 NM_005637)‐NEDD4 (ex14‐29 NM_006154) as well as mutations in the following genes: *IDH1 R109K, AXIN1 A526V, CD36 splice sites 429+2T>C* with the mutation allelic frequencies of 48.17%, 48.26%, and 47.90%, respectively (Figure 2A, B). Loss of *CDKN2A/B* was also identified. Rearrangement of *SS18* was identified by FISH, further corroborating results of the NGS panel (Figure 2C).

Taken together, the overall features were considered to be unclassified malignant cutaneous spindled and epithelioid sarcoma with a novel *SS18::NEDD4* fusion gene. The aggressive clinical nature of this entity, however, was striking. Despite aggressive multimodality treatment approaches, the patient succumbed to her illness within 2 years of presentation.

## 4 DISCUSSION

Our case is the first report of a non‐SS malignant neoplasm identified in the literature to harbor the novel *SS18::NEDD4* fusion gene. Argani et al. recently described the first primary renal SS with positive immunoreactivity for TLE1.^1^ Also, unique to this case was the bland histomorphology defined by a predominantly hypocellular spindle‐cell proliferation within a myxoid stroma, akin to the myxoid variant of myxoid SS. The genomic coordinates of the index case corresponding to the breakpoints in exons 1‐9 of *SS18* and exons 14‐29 of *NEDD4* produce an identical *SS18::NEDD4* fusion to that identified in the primary renal SS.

Although the mechanisms by which the novel *SS18::NEDD4* gene fusion have not been fully elucidated, individual perturbations in SS18‐ and NEDD4‐mediated signaling pathways are recognized for promoting human cancer and tumorigenesis.

The *SS18* gene is ubiquitously expressed in normal human tissue and encodes for the SS18 subunit of mammalian SWI/SNF (BAF) chromatin remodeling complexes which functions to maintain balance in both gene activation and repression.^5^, ^11^, ^12^ The *SS18::SSX* fusion gene results from the replacement of the last eight C‐terminal amino acids of SS18 by 78 C‐terminal amino acids of SSX. The fusion product produces the *SS18::SSX* oncogene which retains the ability of SS18 to incorporate into BAF complexes, however, displacing the BAF47 (SMARCB1/SNF5/INI1) tumor suppressor subunit.^5^

Neural precursor cell‐expressed developmentally downregulated gene 4 (*NEDD4*) is a member of the E3 ubiquitin ligases.^13^ The NEDD4 subfamily proteins are key regulators of cell growth, endocytosis, and modulation of neuromuscular junctions.^14^, ^15^ Recent studies have emerged to reveal the potential role of NEDD4 in human cancer by modulating ubiquitination‐dependent degradation of downstream substrates involved in carcinogenesis and tumorigenesis including IGF‐IR, FGFR1, EGFR, VEGFR2, HER3/Erb3, PTEN, Notch, phosphor‐Akt, c‐Myc, Ras, and mdm2.^14^, ^15^ Increased expressions of *NEDD4* has been identified by immunohistochemical staining in gastric and colorectal cancer, hepatocellular carcinoma, non‐small‐cell lung carcinoma, invasive ductal carcinoma, and endometrial adenocarcinoma.^16^, ^17^, ^18^ However, *NEDD4* rearrangements have not yet been identified in the aforementioned entities.

The spectrum of cutaneous spindle cell malignancies spans the entire gamut from sarcomatoid carcinoma and spindle cell/desmoplastic melanomas to frank sarcomas, with differing prognostic and therapeutic implications. From a purely molecular perspective, we had considered the possibility of SS, but the morphologic phenotype bears no resemblance to SS. SS is an aggressive malignant mesenchymal neoplasm of uncertain origin, characterized by reciprocal t(X,18)(p11;q11) resulting in *SS18::SSX1/2* or *4* in the vast majority of cases. It can present as a monomorphic proliferation of spindle cells or as a biphasic neoplasm with epithelioid differentiation. There are three principal types including: monophasic fibrous type, biphasic, and poorly differentiated type. Histologically, they are composed of sweeping fascicles of spindle cells with prominent nuclear hyperchromasia. Variable collagen deposition can be seen in the monophasic type. Biphasic SS may contain glandular components, and poorly differentiated round cell morphology can be seen in the poorly differentiated type. The immunohistochemical profile demonstrates diffuse positivity for SS18‐SSX and variable reactivity for keratins and/or epithelial membrane antigen.^19^ TLE1 has been regarded as a highly sensitive and relatively specific marker of synovia sarcomas with strong and diffuse positive immunoreactivity. However, the heterogeneity and low specificity of TLE1 expression in non‐SSs should be recognized in the differential diagnosis including epithelioid sarcoma which has shown multifocal positivity in 2 out of 6 cases with 2+ and 3+ staining in a large study of 163 soft tissue tumors.^20^

In the index case, the leading differential diagnosis based on histomorphology alone was epithelioid sarcoma. Epithelioid sarcoma is a relatively rare malignant mesenchymal neoplasm defined by epithelioid cytomorphology and evidence of epithelial differentiation measured by positive keratin expression by immunohistochemistry and loss of INI1 (SMARCB1).^21^ The classic subtype accounts for two‐thirds of epithelioid sarcoma and most commonly occurs in adolescents and young adults arising within the distal extremities, in particular, the hand and fingers. The proximal variant of epithelioid sarcoma exhibits a pleomorphic and anaplastic morphology with aggressive biology and commonly arises within the deep soft tissues of the pelvis, inguinal region, axilla, and flank. Rarely, the spindled histomorphology may dominate and has been reported as fibroma‐like epithelioid sarcoma. Recurrence of epithelioid sarcoma is common and may occur in up to 70% of cases.^22^ Metastasis of epithelioid sarcoma, including distant metastasis to lymph nodes, visceral organs, and skin, can occur in up to 50% of cases and is uniformly fatal.^22^

Given the intimate association of the neoplastic cells with the overlying skin, we considered the possibility of sarcomatoid squamous cell carcinoma. Sarcomatoid squamous cell carcinoma shares overlapping features with sarcomas. However, the growth pattern, lack of background keratinocyte atypia from chronic ultraviolet light exposure, and lack of expression markers such as p40, p63, CK5/6 makes this an unlikely consideration. Furthermore, the genomic profile, lacking chronic ultraviolent light molecular signature, low tumor mutational burden, and the clinical behavior of widespread cutaneous and visceral metastases (and sparing lymph nodes) suggests that the index case was mesenchymal in origin rather than epithelial in nature. Interestingly, our case demonstrated multiple additional genetic aberrations, specifically, single nucleotide variants, SNV, involving *AXIN1 A526V, CD36* splice site 429+2T>C, and *IDH1* R109K; all of which have been identified in the literature. However, the pathological significance of the SNVs in the index case is unknown. Loss of *CDKN2A/B* has been identified in soft tissue sarcomas, B‐cell non‐Hodgkin lymphoma, and astrocytoma.^23^ Unsupervised clustering of transcriptome profiles by RNA‐sequencing in the previously reported case of *SS18::NEDD4* fusion‐positive primary renal SS revealed close grouping with 3 SSs within the database providing further evidence to support the diagnosis.^1^ While clustering analysis was not performed for the index case, we note that the genomic breakpoints for the *SS18* and *NEDD4* genes were different in both cases which may result in different domain compositions of the fusion oncoprotein. Obviously, whether they cluster together or not remains speculative. Importantly, the resultant morphologic and immunophenotypic profile and biologic behavior in our index differs greatly from the previously reported case of SS. This case underscores the significance of molecular pleiotropism and the inherent promiscuity of similar fusion genes that arise in tumors with divergent histotypes and biological behaviors, with increasing clinical use of NGS platform. It is also conceivable that additional factors including epigenetic and stochastic genomic events may play a role.

The detection of a *CDKN2A* deletion has been recognized in a recent study as a potential biomarker for poor prognosis in soft tissue sarcomas, including subtypes such as SS, malignant peripheral nerve sheath tumor, sarcoma NOS, and undifferentiated pleomorphic sarcoma.^24^

Finally, we considered the possibility of spindle cell malignant melanoma with aberrant cytokeratin reactivity. The marked morphologic and immunophenotypic diversity in malignant melanoma is well documented. Indeed, malignant melanomas have been known to express a wide variety of markers including cytokeratin, desmin, smooth muscle actin, amongst others.^25^ However, in the majority of cases, the melanocytic immunophenotypic profile is retained and lost after dedifferentiation to a sarcomatoid phenotype.^25^ In our case, the uniform cytomorphology (lacking a dedifferentiated component) and absence of melanocytic expression greatly diminished this diagnostic consideration.

In summary, we report a case of an unclassified keratin positive cutaneous spindled and epithelioid sarcoma, resembling epithelioid sarcoma, and harboring the *SS18‐NEDD4* fusion with a very aggressive clinical behavior in a 60‐year‐old female. Our case displayed an aggressive and fatal clinical course different from that of the previously reported primary renal SS harboring an identical *SS18‐NEDD4* fusion gene. The mechanisms are not yet clear. However, it is conceivable that the constellation of multiple genetic hits including *CDKN2A* mutation may have played a role in the aggressive biology. Future studies are warranted to further characterize this novel entity.

## AUTHOR CONTRIBUTIONS

**Obiajulu Hans Iwenofu**, conceived the project. **Ashley Patton**, **Steve Oghumu**, and **Obiajulu Hans Iwenofu** provided essential tools/data. AP & OHI wrote the paper. All authors approved the final manuscript.

## CONFLICT OF INTEREST

The authors declare no conflicts of interest.

## Data Availability

Data sharing not applicable to this article as no datasets were generated or analysed during the current study.

## References

[1] Argani P , Zhang L , Sung YS , et al. Novel SS18‐NEDD4 gene fusion in a primary renal synovial sarcoma. Genes Chromosomes Cancer. 2020;59(3):203‐208. doi:10.1002/gcc.22814 31595587 PMC7086378

[2] Storlazzi CT , Mertens F , Mandahl N , et al. A novel fusion gene, SS18L1/SSX1, in synovial sarcoma. Genes Chromosomes Cancer. 2003;37(2):195‐200. doi:10.1002/gcc.10210 12696068

[3] Gazendam AM , Popovic S , Munir S , Parasu N , Wilson D , Ghert M . Synovial Sarcoma: A Clinical Review. Curr Oncol Tor Ont. 2021;28(3):1909‐1920. doi:10.3390/curroncol28030177 PMC8161765 34069748

[4] Baranov E , McBride MJ , Bellizzi AM , et al. A novel SS18‐SSX fusion‐specific antibody for the diagnosis of synovial sarcoma. Am J Surg Pathol. 2020;44(7):922‐933. doi:10.1097/PAS.0000000000001447 32141887 PMC7289668

[5] Kadoch C , Crabtree GR . Reversible disruption of mSWI/SNF (BAF) complexes by the SS18‐SSX oncogenic fusion in synovial sarcoma. Cell. 2013;153(1):71‐85. doi:10.1016/j.cell.2013.02.036 23540691 PMC3655887

[6] Bishop JA , Weinreb I , Swanson D , et al. Microsecretory adenocarcinoma: a novel salivary gland tumor characterized by a recurrent MEF2C‐SS18 fusion. Am J Surg Pathol. 2019;43(8):1023‐1032. doi:10.1097/PAS.0000000000001273 31094920

[7] Zhang M , Mao D , Zhang W . The pathogenic role of MEF2D‐SS18 fusion gene in B‐cell acute lymphoblastic leukemia. Biochem Biophys Res Commun. 2018;496(4):1331‐1336. doi:10.1016/j.bbrc.2018.02.013 29408457

[8] Antonescu CR , Agaram NP , Sung YS , Zhang L , Dickson BC . Undifferentiated round cell sarcomas with novel SS18‐POU5F1 fusions. Genes Chromosomes Cancer. 2020;59(11):620‐626. doi:10.1002/gcc.22879 32557980 PMC8115304

[9] He J , Abdel‐Wahab O , Nahas MK , et al. Integrated genomic DNA/RNA profiling of hematologic malignancies in the clinical setting. Blood. 2016;127(24):3004‐3014. doi:10.1182/blood-2015-08-664649 26966091 PMC4968346

[10] Frampton GM , Fichtenholtz A , Otto GA , et al. Development and validation of a clinical cancer genomic profiling test based on massively parallel DNA sequencing. Nat Biotechnol. 2013;31(11):1023‐1031. doi:10.1038/nbt.2696 24142049 PMC5710001

[11] D'Arcy P , Maruwge W , Ryan BA , Brodin B . The oncoprotein SS18‐SSX1 promotes p53 ubiquitination and degradation by enhancing HDM2 stability. Mol Cancer Res MCR. 2008;6(1):127‐138. doi:10.1158/1541-7786.MCR-07-0176 18234968

[12] McBride MJ , Kadoch C . Disruption of mammalian SWI/SNF and polycomb complexes in human sarcomas: mechanisms and therapeutic opportunities. J Pathol. 2018;244(5):638‐649. doi:10.1002/path.5042 29359803 PMC6755911

[13] Yang B , Kumar S . Nedd4 and Nedd4‐2: closely related ubiquitin‐protein ligases with distinct physiological functions. Cell Death Differ. 2010;17(1):68‐77. doi:10.1038/cdd.2009.84 19557014 PMC2818775

[14] Wang X , Trotman LC , Koppie T , et al. NEDD4‐1 is a proto‐oncogenic ubiquitin ligase for PTEN. Cell. 2007;128(1):129‐139. doi:10.1016/j.cell.2006.11.039 17218260 PMC1828909

[15] Wang ZW , Hu X , Ye M , Lin M , Chu M , Shen X . NEDD4 E3 ligase: functions and mechanism in human cancer. Semin Cancer Biol. 2020;67(Pt 2):92‐101. doi:10.1016/j.semcancer.2020.03.006 32171886

[16] Bellet MM , Piobbico D , Bartoli D , et al. NEDD4 controls the expression of GUCD1, a protein upregulated in proliferating liver cells. Cell Cycle Georget Tex. 2014;13(12):1902‐1911. doi:10.4161/cc.28760 PMC4111753 24743017

[17] Jung S , Li C , Jeong D , et al. Oncogenic function of p34SEI‐1 via NEDD4‐1‐mediated PTEN ubiquitination/degradation and activation of the PI3K/AKT pathway. Int J Oncol. 2013;43(5):1587‐1595. doi:10.3892/ijo.2013.2064 23970032

[18] Zhang Y , Goodfellow R , Li Y , et al. NEDD4 ubiquitin ligase is a putative oncogene in endometrial cancer that activates IGF‐1R/PI3K/Akt signaling. Gynecol Oncol. 2015;139(1):127‐133. doi:10.1016/j.ygyno.2015.07.098 26193427 PMC4586405

[19] Foo WC , Cruise MW , Wick MR , Hornick JL . Immunohistochemical staining for TLE1 distinguishes synovial sarcoma from histologic mimics. Am J Clin Pathol. 2011;135(6):839‐844. doi:10.1309/AJCP45SSNAOPXYXU 21571956

[20] Kosemehmetoglu K , Vrana JA , Folpe AL . TLE1 expression is not specific for synovial sarcoma: a whole section study of 163 soft tissue and bone neoplasms. Mod Pathol. 2009;22(7):872‐878. doi:10.1038/modpathol.2009.47 19363472

[21] Heenan PJ , Quirk CJ , Papadimitriou JM . Epithelioid sarcoma. A diagnostic problem. Am J Dermatopathol. 1986;8(2):95‐104. doi:10.1097/00000372-198604000-00002 3717528

[22] Levy A , Le Péchoux C , Terrier P , et al. Epithelioid sarcoma: need for a multimodal approach to maximize the chances of curative conservative treatment. Ann Surg Oncol. 2014;21(1):269‐276. doi:10.1245/s10434-013-3247-4 24046109

[23] Reis GF , Pekmezci M , Hansen HM , et al. CDKN2A loss is associated with shortened overall survival in lower‐grade (World Health Organization Grades II‐III) astrocytomas. J Neuropathol Exp Neurol. 2015;74(5):442‐452. doi:10.1097/NEN.0000000000000188 25853694 PMC4397174

[24] Bui NQ , Przybyl J , Trabucco SE , et al. A clinico‐genomic analysis of soft tissue sarcoma patients reveals CDKN2A deletion as a biomarker for poor prognosis. Clin Sarcoma Res. 2019;9:12. doi:10.1186/s13569-019-0122-5 31528332 PMC6739971

[25] Compton LA , Murphy GF , Lian CG . Diagnostic immunohistochemistry in cutaneous neoplasia: An update. Dermatopathol Basel Switz. 2015;2(1):15‐42. doi:10.1159/000377698 PMC4816435 27047932

